# Frequency dependence of power and its implications for contractile function of muscle fibers from the digital flexors of horses

**DOI:** 10.14814/phy2.12174

**Published:** 2014-10-07

**Authors:** Michael T. Butcher, John E.A. Bertram, Douglas A. Syme, John W. Hermanson, P. Bryant Chase

**Affiliations:** 1 Department of Biological Sciences, Youngstown State University, Youngstown, Ohio, USA; 2 Department of Cell Biology and Anatomy, Faculty of Medicine, University of Calgary, Calgary, Alberta, Canada; 3 Department of Biological Sciences, University of Calgary, Calgary, Alberta, Canada; 4 Department of Biomedical Sciences, College of Veterinary Medicine, Cornell University, Ithaca, New York, USA; 5 Department of Biological Science, Florida State University, Tallahassee, Florida, USA

**Keywords:** Absorption, deep digital flexor, skinned fiber, superficial digital flexor, workloop

## Abstract

The digital flexors of horses must produce high force to support the body weight during running, and a need for these muscles to generate power is likely limited during locomotion over level ground. Measurements of power output from horse muscle fibers close to physiological temperatures, and when cyclic strain is imposed, will help to better understand the in vivo performance of the muscles as power absorbers and generators. Skinned fibers from the deep (DDF) and superficial (SDF) digital flexors, and the soleus (SOL) underwent sinusoidal oscillations in length over a range of frequencies (0.5–16 Hz) and strain amplitudes (0.01–0.06) under maximum activation (pCa 5) at 30°C. Results were analyzed using both workloop and Nyquist plot analyses to determine the ability of the fibers to absorb or generate power and the frequency dependence of those abilities. Power absorption was dominant at most cycling frequencies and strain amplitudes in fibers from all three muscles. However, small amounts of power were generated (0.002–0.05 Wkg^−1^) at 0.01 strain by all three muscles at relatively slow cycling frequencies: DDF (4–7 Hz), SDF (4–5 Hz) and SOL (0.5–1 Hz). Nyquist analysis, reflecting the influence of cross‐bridge kinetics on power generation, corroborated these results. The similar capacity for power generation by DDF and SDF versus lower for SOL, and the faster frequency at which this power was realized in DDF and SDF fibers, are largely explained by the fast myosin heavy chain isoform content in each muscle. Contractile function of DDF and SDF as power absorbers and generators, respectively, during locomotion may therefore be more dependent on their fiber architectural arrangement than on the physiological properties of their muscle fibers.

## Abstract (precis)

Equine digital flexor muscles fibers have a relatively large capacity for energy absorption. This physiological property of their muscle fibers may be important to the function of these specialized distal limb muscles during locomotion.

## Introduction

Contractile performance of muscles is a functional extension of the contractile properties of their muscle fibers, which are in turn determined to a large extent by the myosin heavy chain (MHC) isoforms expressed in single fibers (Schiaffino and Reggiani 1996). Isometric tension (*P*_0_), shortening velocity (*V*_0_), and power output (*W*) are properties commonly measured to describe the contractile abilities of skinned muscle fibers (Sweeney et al. 1986; Bottinelli et al. 1996, 1999; Pellegrino et al. 2003; Butcher et al. 2010). Such measures provide valuable insight into the capacity of muscle to contribute to locomotion either through providing limb support or as a source (or sink) of mechanical work and power, both proposed as important functions for locomotion in horses (Wilson et al. 2001). Conventionally, power output is determined from the force‐velocity relationship in single, skinned fibers (Seow and Ford 1991; Bottinelli et al. 1996; Kohn and Noakes 2013), where force‐velocity data are fit with a hyperbolic curve (Hill 1938) and peak power is derived from the product of fiber force and shortening velocity. Force‐velocity properties have been previously measured in horse muscle fibers at sub‐physiological temperatures (Rome et al. 1990) and therefore, power output derived from these data (West et al. 2013) make extrapolation to in vivo performance difficult. As such, measurements of mechanical power from horse muscle fibers close to physiological temperatures, and when cyclic strain is imposed on the fibers as occurs during running (Biewener and Roberts 2000), are needed to better understand the structure and function of their muscles to either produce force or generate power during locomotion.

Maximally activated muscle fibers can be subjected to sinusoidal oscillations at varying cycling frequencies to determine mechanical work (Josephson 1985). These “workloop” analyses were originally utilized in studies of insect flight muscle (Machin and Pringle 1959, 1960; Tregear 1983), and have since been adapted to study conditions of mechanical work and power output in limb muscles, especially how these properties relate to performance during locomotion (Barclay 1994; Askew et al. 1997, 2001; Syme and Josephson 2002; Syme et al. 2005). While workloop analyses are often employed on intact limb muscles or living fascicles as a means to measure power generation by muscle, sinusoidal oscillations on single, skinned fibers can also be used as a high resolution method to study details of the cross‐bridge cycle and its rate constants (Kawai and Brandt 1980; Zhao and Kawai 1994), to understand the material viscoelastic properties of muscle and the circumstances under which muscles may act as either energy absorbers or generators. In such analyses, Nyquist plots (Kawai and Schachat 1984), where the viscous modulus is plotted as a function of the elastic modulus, are used to measure the magnitudes of both moduli, their dependence on oscillation frequency, and how it relates to elastic storage versus viscous loss. Values of both moduli (determined at different cycling frequencies) also form distinct relationships that are characteristic for “slow” and “fast” fiber types (Kawai and Brandt 1980), and are highly dependent on experimental temperature (Zhao and Kawai 1994; Wang and Kawai 2001). Thus, these types of studies would be useful to evaluate the contractile properties of horse muscles that contain both slow and fast MHC isoform fibers and must produce high force to support the body weight, and resist large strains their long tendons experience with each footfall (Butcher et al. 2007).

Nyquist plots from fast fiber types (e.g., rabbit psoas; MHC‐2X) in particular, show three distinct exponential processes of cross‐bridge cycling, A, B, and C (Kawai and Schachat 1984), that can be used to infer muscle function. Process A is a *low frequency‐exponential advance* where muscle fibers absorb work; Process B is a *medium frequency‐exponential delay* where fibers generate power; and Process C is a *high frequency‐exponential advance* where fibers again absorb work (Kawai and Brandt 1980). These three processes arise from chemo‐mechanical reactions of actively cycling cross‐bridges (Kawai and Schachat 1984), thus they are absent from relaxed and rigor fibers. Process B of the Nyquist plot (the loop) is also known as the oscillatory work component (Pringle 1967; Wang and Kawai 1997). Active muscle will show one clockwise loop approximately centered on the elastic modulus axis, with the diameter of the loop being the magnitude of the process (Kawai and Brandt 1980). At low strain amplitudes the polarity of Process B is uniquely negative due to a negative phase shift (*ϕ*) (i.e., length leads force maximum in time), thus the *ϕ* of the complex modulus is an indicator of the direction of *net* work flow. Maximum oscillatory power occurs at some characteristic frequency *b* where the viscous modulus (component of work that is absorbed) is most negative (Kawai and Schachat 1984). Therefore, small amplitudes of strain correspond to length changes in the range of attached cross‐bridge sliding and power generation, while large strain amplitudes exceed the cross‐bridge motion range and lead to power absorption.

The aim of this study was to determine the capacity for work and power in horse muscle fibers near physiological temperature (30°C) via sinusoidal length oscillations using both workloop and Nyquist plot analyses. Fiber work and power data will build on our previous analyses of MHC isoform fiber type and fiber contractile properties in the forelimb deep digital flexor (DDF) and superficial digital flexor (SDF) muscle fibers (Butcher et al. 2010), and how these physiological properties relate to muscle architecture and contractile function during locomotion (Butcher et al. 2007, 2009). Briefly, the DDF (short compartment) shortens to flex the digit during running and the SDF produces high force while lengthening to support the weight of the animal (Butcher et al. 2007, 2009). This differential contractile function of having some distal limb muscles that perform mechanical work while others contract isometric (or eccentrically) to resist muscle‐tendon unit strain is common in running mammals (Biewener and Roberts 2000), and reduces metabolic energy consumption during locomotion. Based on these different in vivo functions, it is hypothesized that power capacity (absorptive and generative) in DDF and SDF will correspond with differences in their respective distributions of fast MHC‐2A isoform fibers (Butcher et al. 2007). It is predicted that faster DDF fibers will have a higher oscillatory work and power output, while slower SDF fibers are expected to have an inherently greater capacity for energy absorption. The findings of this study will improve our understanding of structure/function and evolution of specialized distal limb muscles in horses.

## Materials and Methods

### Ethical approval

All protocols for harvesting muscle tissue from horses were in accordance with the policies and standards of the Cornell University, College of Veterinary Medicine, Equine Hospital and Institutional Animal Care and Use Committee (IACUC) approved guidelines and protocols. Fiber contractile properties experiments were performed according to protocols approved by the Florida State University (FSU) IACUC.

### Muscle sampling, skinned fiber preparation, and experiment solutions

Two adult horses (one female Thoroughbred and one female Appaloosa) from our previous study of contractile properties and MHC fiber type in DDF and SDF muscles (Butcher et al. 2010) provided muscle tissue for this study. The age and body mass of the horses were 21 years and 482.7 kg (Thoroughbred) and 14 years and 454.5 kg (Appaloosa). Immediately following euthanasia, the DDF (short humeral head) and SDF muscles of the forelimb, and the soleus (SOL) muscle of the hindlimb were removed and 2–3 small fiber fascicles were dissected from the mid‐belly region of each muscle and tied to Teflon^®^ strips at resting length.

Freshly dissected muscle fascicles were prepared for mechanical experimentation using published methods (Chase and Kushmerick 1988; Chase et al. 1994) and also described in our previous report (see ref. Butcher et al. 2010). Relaxing (pCa 9) and maximally activating (pCa 5) stock solutions were prepared as previously described (Chase and Kushmerick 1988; Butcher et al. 2010). Briefly, the composition of the solutions was (in mmol/L): 5 MgATP, 1 Pi, 10 EGTA, 15 PCr (CP), 100 K^+^ plus Na^+^, 3 Mg^2+^, 50 MOPS, 1 dithiothreitol (DTT), and 1 mg/mL creatine kinase (CK; 260 U mL^−1^). Ca^2+^ concentrations in the stock solutions were adjusted by adding appropriate amounts of Ca(acetate)_2_. A rigor solution (pCa 5) was prepared with a similar composition except lacking MgATP, CP, and CK. The pH was adjusted to 7.0 for all solutions at 12°C, and the ionic strength was 0.18 M and was adjusted with Tris and acetate (Butcher et al. 2010). Experimental solutions were used at 10°C and 30°C with no further adjustments to pH and ionic strength for minor changes with temperature as have been described (Bottinelli et al. 1996; Stienen et al. 1996). DTT and CK were added to relaxing and activating solutions on the day of each experiment. DTT only was added to rigor solutions before an experiment.

### Experimental apparatus

The experimental apparatus (Chase et al. 1994, 1998; Martyn et al. 1994) and the force transducer (Butcher et al. 2010) have been described in detail previously. Relaxing, activating, and rigor solutions were held in anodized aluminum wells (200 mL) with glass coverslip (no. 1 thickness) bottoms. The temperature of the wells was measured with a k‐type thermocouple and was maintained within 1°C at either 10°C or 30°C depending on the experiment (ATR‐4 Adaptable Thermoregulator, Quest Scientific, North Vancouver, BC, Canada). One well was maintained at 10°C and contained relaxing solution (pCa 9) used for measurement of fiber dimensions and relaxation of fibers between length oscillation trials. The other well was maintained at 30°C and contained activating solution (pCa 5) used for measurement of contractile properties. Force and length signals were digitized using custom data acquisition software (NEWDAC; refs. Chase et al. 1994, 1998) on a PC‐based system with a DT2831‐G data acquisition board (Data Translation, Marlboro, MA). To avoid high frequency aliasing, all force and length signals were low pass filtered (4th order Bessel filter *f*_c _≤ 40% of the sampling rate) using a CyberAmp 380 (Axon Instruments, Foster City, CA) before digitization (Regnier et al. 1996). The sampling rate varied with oscillation frequency to further avoid aliasing such that 10 complete cycles were acquired at all but the lowest frequencies, with at least 100 data samples per mechanical oscillation (Regnier et al. 1996). Stability of fiber structure and mechanical properties during activations were maintained by Brenner cycling (Brenner 1983). Fiber striation pattern was continuously monitored during experiments by a CCD camera (model XR‐77, Sony Electronics Corp., Tokyo, Japan).

### Experimental protocol

All mechanical measurements were made at both 10°C and 30°C. Isometric fiber length (*L*_0_), fiber diameter and sarcomere length (*L*_s_) were measured in relaxing conditions as previously described (Butcher et al. 2010). *L*_0_ was monitored during the experiment and commonly *L*_0_ and fiber diameter were re‐measured after the experiment. *L*_s_ was initially set to 2.5 *μ*m in relaxing conditions, and *L*_0_ was then adjusted to minimize passive tension; under these conditions mean (±SE) *L*_s_ was 2.4 ± 0.05 *μ*m (*n = *9) for DDF fibers, 2.4 ± 0.04 *μ*m (*n = *8) for SDF fibers, and 2.4 ± 0.06 *μ*m (*n = *8) for SOL fibers. All other dimensional data of relaxed DDF, SDF, and SOL fibers are presented in Table 1.

**Table 1.** Means ± SE of fiber dimensions used in normalization of fiber oscillatory work and power

Muscle	Active fiber length (mm)	Fiber diameter (*μ*m)	Fiber CSA (*μ*m^2^)	Fiber mass (mg)	*n*
DDF	1.21 ± 0.08	101 ± 6.41	8329 ± 978	0.009 ± 0.001	11
SDF	1.06 ± 0.06	79.9 ± 3.62	5132 ± 496	0.006 ± 0.001	12
SOL	1.01 ± 0.05	47.8 ± 2.43	1837 ± 199	0.002 ± 0.001	10

**Figure 1.**
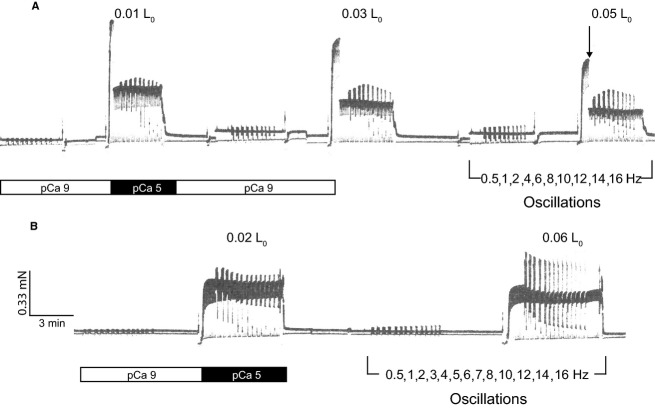
Example recordings of force versus time of single, skinned SDF fibers at 30°C illustrating the measurement protocol. Data were obtained during alternating periods of relaxation (pCa 9) and maximal activation (pCa 5). Brief periods of unloaded shortening [“Brenner cycling” (ref. Butcher et al. 2010): see Materials and Methods] are evident as periodic force transients (every 5 sec) throughout the entirety of both records. (A) Fibers underwent sinusoidal length oscillations at three strain amplitudes (0.01, 0.03, 0.05 examples shown) and over a range of cycling frequencies (0.5–16 Hz) at each strain. (B) Alternatively, for some experiments fibers underwent sinusoidal length oscillations at two strain amplitudes (high and low) over a more extensive set of cycling frequencies (16 Hz repeated twice in the force records). Scale bars indicate the same force (mN) and time (min) magnitudes for each recording. ↓ Changes in gain. Note the change in fiber force between pCa9 and pCa5 during sinusoidal length oscillations.

Changes in fiber force (∆ P) and fiber length (∆ FL) were measured in relaxing, maximal activating, and rigor conditions when imposing a series of sinusoidal oscillations on muscle length (see ref. Kawai and Brandt 1980). Prior to each series of oscillations, measurements of fiber force (*P*) were made during the steady‐state period before a Brenner cycle (Martyn et al. 1994) to establish force scales for data analysis. P was normalized to fiber cross‐sectional area (CSA) yielding force as mN mm^−2^, where CSA was calculated from mean fiber diameter assuming circular geometry. The maximum isometric tension (*P*_0_) was determined at pCa 5. Beginning in relaxing conditions, single fibers underwent sinusoidal length oscillations over a range of frequencies encompassing physiologically relevant frequencies (0.5–16 Hz) and peak‐to‐peak strain amplitudes (0.01–0.06 *L*_0_). Measurements of ∆ *P* and ∆ FL at each strain amplitude studied were made at sequentially higher frequencies in the following order: 0.5, 1, 2, 3, 4, 5, 6, 7, 8, 10, 12, 14, 16 Hz. Experiments proceeded by alternating between relaxing (pCa 9, 10°C) and maximal activation (pCa 5, 30°C) conditions for a series of oscillations at the same strain amplitude, with 1–2 min relaxation of fibers between series (Fig. 1). Similar temperature jump methods (Pate et al. 1994) have been used in previous studies of power in single, skinned fibers (Knuth et al. 2006; West et al. 2013). Strain amplitude was then increased and the process repeated, until all frequency/strain/pCa combinations had been completed. Experiments ended with the same series of oscillations in rigor; induced sequentially in two rigor solutions to obtain a “high rigor” state (Kawai and Brandt 1976). Measurements of ∆ *P* and ∆ FL in rigor were made only in the second rigor solution and without Brenner cycling.

For each fiber, measurements were made at each of three strain amplitudes, either 0.01, 0.03, 0.05 *L*_o_ or 0.02, 0.04, 0.06 *L*_0_, and in several experiments, measurements were made at only two strain amplitudes (0.01 and 0.06 *L*_0_ or 0.02 and 0.06 *L*_0_) (Fig. 1). This protocol was used to limit fiber degradation and force decline between the first and last series of sinusoidal oscillations, and to facilitate comparison of results across strains and frequencies. However, the similarity of force during first and last activations indicated that fiber structure and function remained quite stable throughout the protocols and so we do not distinguish between experiments with two versus three strain amplitudes tested. Force at the end of experiments averaged 94.8% of that at the beginning for DDF fibers, 98.9% for SDF fibers, and 88.7% for SOL fibers at 10°C; and was 73.6% for DDF fibers, 83.7% for SDF fibers and 91.3% for SOL fibers at 30°C. A minimal stability of 70% was considered acceptable, and fibers with a lower stability were excluded from the analysis. Similar criteria were used for other fiber studies at 22°C (Stienen et al. 1996).

### Data analysis

Initial data analysis involved setting force and length scales for all data records of ∆ P (in mN) and ∆ FL (in *μ*m) from each series of oscillations using custom software (Fiber Analysis) (Chase et al. 1994, 1998). Additional analysis of sinusoidal data was conducted using two routines.

#### Workloop analysis

*Net* oscillatory work (Machin and Pringle 1959; Pringle 1967; Kawai and Brandt 1980; Josephson 1985) was calculated using customized software written in LabVIEW (National Instruments, Austin, TX) to integrate the area enclosed by the loop formed by plotting ∆ P (in N) as a function of ∆ FL (in m). The area enclosed by the loop is equal to the net mechanical energy either absorbed or generated per cycle of length oscillation (Kawai and Brandt 1980). Average *net* oscillatory work per cycle (*J*_avg_, in Joules) was determined from total *net* work (cumulative across all cycles analyzed) divided by the number of cycles analyzed (which depended on oscillation frequency). Average *net* oscillatory power (*W*_avg_, in W kg^−1^ muscle fiber) was determined from *J*_avg_ multiplied by oscillation frequency and normalized to fiber mass. Fiber mass was calculated as *L*_0 _× fiber CSA (assuming circular geometry) multiplied by a density of 1.05 g cm^−3^. *W*_avg_ for DDF, SDF, and SOL fibers were averaged for each combination of oscillation frequency and strain amplitude (0.01–0.06) tested in maximally activated fibers (pCa 5) at 30°C.

#### Nyquist analysis

**Figure 2.**
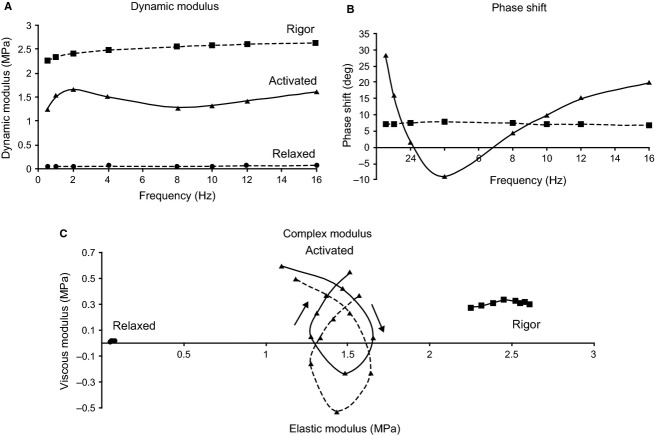
Example of Nyquist analysis from dynamic modulus (*D*_M_) and phase shift (*ϕ*) obtained from sinusoidal length oscillations of a single, skinned SDF fiber at 0.01 strain and 30°C. (A) *D*_M_ versus frequency comparing maximally activating (pCa 5), relaxing (pCa 9) and rigor (pCa 5 with no ATP) conditions. At pCa 9, *D*_M_ is low and does not change with frequency, whereas *D*_M_ is high and increases slightly with frequency in rigor fibers. (B) *ϕ* versus frequency comparing maximally activating (▲) and rigor (■) conditions. *ϕ* changes minimally with frequency in rigor fibers. (C) Nyquist plots showing the complex modulus formed by plotting the viscous modulus (*V*) as a function of the elastic modulus (*E*) with cycling frequency as the intervening parameter (individual data points). Nyquist plots loop clockwise with increasing frequency. Rigor corrected plots (dashed line) shift down on the *y* axis relative to uncorrected plots (solid line). Relaxed fibers exhibit low viscous/elastic moduli, while rigor fibers have high viscous/elastic moduli. Only actively contracting fibers produce characteristic Nyquist plots of fibers exhibiting oscillatory work over a range of frequencies.

Dynamic stress modulus (*D*_M_, in MPa) and phase shift (*ϕ*, in degrees) were calculated (Wang and Kawai 1996, 1997) using a Fast Fourier Transform (FFT) analysis (Fiber Analysis software) on records of ∆ *P*_0_ (mN mm^−2^) and ∆ FL (% strain) from each series of oscillation frequencies and strain amplitudes. For each oscillation series for each fiber, *ϕ* measured in rigor at the same strain amplitude and temperature (Kawai and Brandt 1980) was subtracted from activated fiber *ϕ* to yield *ϕ*_c_, the phase shift corrected for lag introduced by the electronic hardware and fiber end compliance. This phase shift was ~9° for all fibers and trials, with no systematic difference among DDF, SDF, and SOL fiber experiments. *D*_M_ and *ϕ*_c_ (shown as Bode plots in Fig. 2A and B) were used to construct Nyquist plots, representing the complex modulus *Y*_M_(ƒ), defined as a ratio of stress change to strain change in the frequency (ƒ) domain (Wang and Kawai 1996, 1997). The real (in‐phase) component of *Y*_M_(ƒ) is the elastic modulus [*E *= *D*_M_.cos(*ϕ*_c_)]. The imaginary (90° out‐of‐phase) component of *Y*_M_(ƒ) is the viscous modulus [*V *= *D*_M_.sin(*ϕ*_c_)] (Kawai and Brandt 1980; Murphy et al. 1996). *V* was then plotted as a function of *E* (Fig. 2C), with each point representing measurements at a different oscillatory frequency (see Figs. 1, 2A and B). Nyquist plot data were averaged (2–3 fibers per muscle) at 0.01 strain amplitude in maximally activated fibers (pCa 5, 30°C). Plots of D_M_ and *ϕ*_c_ values as a function of oscillation frequency were evaluated for characteristic minima (downward inflection) along the curves and the frequencies at which the minima occurred were compared. Similarly, individual Nyquist plots from each muscle were evaluated qualitatively for shape and compared for frequencies at which the muscles performed (rather than absorbed) work (i.e., when *V* is negative). The characteristic frequency of maximum oscillatory work (minimum value of *V*) was labeled *b* on Nyquist plots following established conventions (Kawai and Schachat 1984).

A strain amplitude of 0.01 corresponds to a cross‐bridge oscillation of ~12 nm, assuming a sarcomere length of 2.5 *μ*m and no compliance in the myofilaments. Sinusoidal analysis is typically conducted at strain amplitudes of 0.0025–0.0050 *L*_0_ for high resolution study of cross‐bridge steps and associated rate constants (Zhao and Kawai 1994; Wang and Kawai 1996, 1997), with a strain of 0.005 corresponding to oscillation of approximately 6 nm per *half‐sarcomere*. Myosin II step size (i.e., power stroke) is less than 15 nm (Finer et al. 1994) and therefore equivalent to about 0.01 strain. With regard to experimental temperature, at temperatures ≤15°C Nyquist plots of muscle fibers deviate from the typical shape showing compression of the loop at the abscissa (Wang and Kawai 2001). This effect is expected to be more extreme for MHC‐1 fibers which show greater sensitivity to temperature. For these reasons, Nyquist plot analysis could only performed on maximally activated fibers (pCa 5) at 30°C and not at strains in excess of 0.01 (some strain will be absorbed by the actin filaments and other sources of compliance). A final consideration is that quantitative Nyquist plot analysis assumes linearity, unlike workloop analysis. Linearity is a function of strain amplitude (Machin and Pringle 1960). At strains ≤0.0025, force response is linear and its time course is described adequately by *sine* and *cosine* functions of the same periodicity, validating the sinusoidal technique. At strain amplitudes up to 0.005 *L*_0_, nonlinearity of force has been shown to be 2.0% (at worst), again justifying a linear analysis (Kawai 1986). However, at higher strain amplitudes used in this study, the relationship between force and length changes becomes more non‐linear (Kawai and Brandt 1980). Therefore, the Nyquist data were specifically used to assess the effects of cycle frequency on the relative capacity of muscle fibers to absorb/perform work at low strain, but not the amount of work per se, while workloop analysis was chosen as the preferred method for calculation of *net* oscillatory work and power (*W*_avg_) at all strain amplitudes tested.

### Statistical analysis

All data reported in tables are expressed as mean ± SE. Statistical significance of the differences among means of minimum (oscillatory power absorption) and maximum *W*_avg_ (oscillatory power generation) of maximally activated (pCa 5, 30°C) fibers for each muscle and strain amplitude were assessed by 1‐way ANOVA followed by Holm‐Sidak multiple comparison tests. No statistical testing was performed on Nyquist plot data. Statistical significance for all tests was accepted at *P *≤**0.05.

## Results

### Workloops

**Figure 3.**
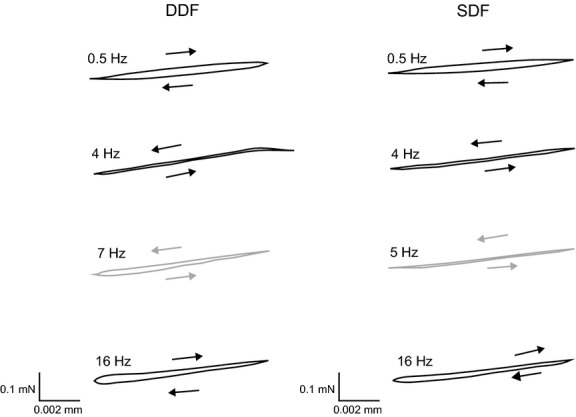
Workloops from maximally activated (pCa 5) DDF (left panels) and SDF (right panels) fibers over a range of sinusoidal frequencies at 0.01 strain and 30°C. Counterclockwise rotation indicates work production and clockwise rotation indicates energy absorption. Left: DDF loops at 0.5 Hz and 16 Hz rotate in a clockwise direction (black arrows) and loops at 4 Hz and 7 Hz rotate in a counterclockwise direction (grey arrows). Right: SDF loops at 0.5 Hz and 16 Hz rotate in a clockwise direction (black arrows) and loops at 4 Hz and 5 Hz rotate in a counterclockwise direction (grey arrows). Number of cycles: 0.5 Hz, *n *=**1; 4–16 Hz, *n *=**9. Scale bars indicate force ∆ P (mN) and length ∆ FL (mm) magnitudes.

Figure 3 shows representative workloops from maximally activated DDF and SDF fibers over a range of oscillation frequencies at 0.01 L_0_ strain amplitude. For low strain amplitudes (0.01 and 0.02 *L*_0_) and the lowest cycling frequencies (0.5, 1–2 Hz), loops had a characteristic elliptical shape and were clockwise in direction (i.e., power absorption). As cycling frequency increased throughout the intermediate range (3–7 Hz) the shape of the loops became compressed, but with a slight opening when fiber length was short (length within an oscillation cycle), and were counterclockwise (i.e., power generation). The upper and lower bounds of the intermediate range of cycling frequencies where power was generated showed variability between DDF and SDF fibers, between individual fiber experiments for each muscle, and also varied with strain amplitude. With further increases in cycling frequency (≥8 Hz), loops opened when fiber length was short and remained more closed when fiber length was long, and were once again clockwise in direction. For strain amplitudes ≥ 0.03 *L*_0_ (Table 2), loops for maximally activated DDF and SDF fibers were all clockwise and relatively open (power absorption) over the range of cycling frequencies tested.

**Table 2.** Grand means ± SE of maximum net oscillatory power measured by workloop analysis from fibers subject to sinusoidal oscillations and maximal activation (pCa 5; 30°C) for the DDF, SDF, and SOL

Strain	DDF				SDF				SOL			
	Power (W kg^−1^)	*n* (cycles)	Frequency ^ƒ^ (Hz)	*n*	Power (W kg^−1^)	*n* (cycles)	Frequency ^ƒ^ (Hz)	*n*	Power (W kg^−1^)	*n* (cycles)	Frequency ^ƒ^ (Hz)	*n*
0.01	0.05 ± 0.00^1^	9	7	1	0.02 ± 0.01^1^	27	4	3	−0.01 ± 0.01	3	0.5	3
0.02	0.00 ± 0.04	18	6	2	−0.05 ± 0.02	4	0.5	4	−0.02 ± 0.01	3	0.5	3
0.03	−0.20 ± 0.08	2	0.5	2	−0.12 ± 0.02	3	0.5	3	−0.11 ± 0.07	2	0.5	2
0.04	−0.21 ± 0.07	3	0.5	3	−0.14 ± 0.02	2	0.5	2	−0.08 ± 0.00	2	0.5	2
0.05	−0.37 ± 0.14	2	0.5	2	−0.28 ± 0.05	3	0.5	3	−0.20 ± 0.10	2	0.5	2
0.06	−0.38 ± 0.10	4	0.5	4	−0.28 ± 0.06	3	0.5	3	−0.17 ± 0.01	3	0.5	3

ƒ frequency at which most power is generated.

*n* (cycles) is the total number of cycles per fiber over which oscillatory power was measured and averaged (*W*_avg_)

*n* is the number of fiber experiments

^1^ Significantly different from slow SOL at *P *<**0.05.

**Figure 4.**
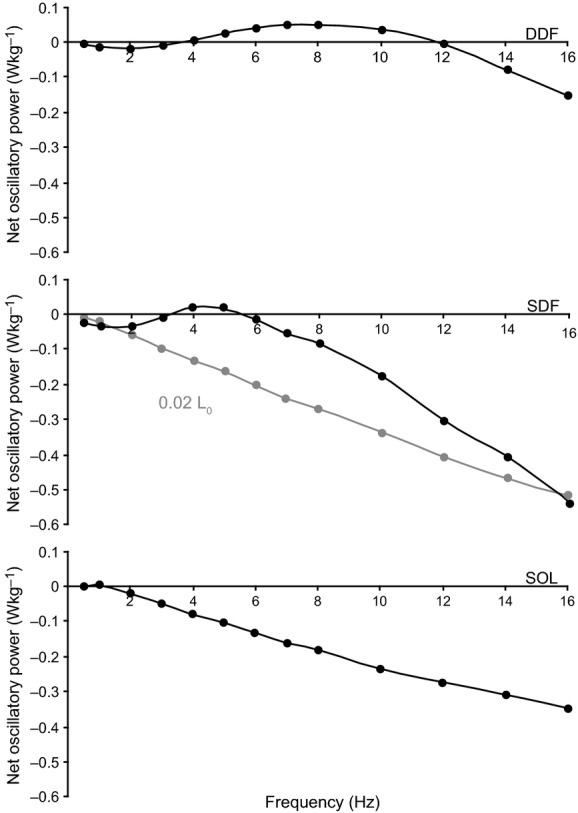
Maximum *net* oscillatory power versus cycling frequency for DDF (top), SDF (middle), and SOL (bottom) fibers at 0.01 strain and 30°C. Results at 0.02 strain (grey curve) are also included for SDF fibers (middle). Oscillatory power was determined by integration of the area within the workloops. Representative data from a single fiber for each muscle is shown.

Means (± SE) of maximum net W_avg_ for maximally activated fibers are presented in Table 2. At 0.01 strain, DDF and SDF fibers generated a small amount of power. The difference between maximum *W*_avg_ of DDF and SDF fibers was not significant (*P *=**0.07), however maximum *W*_avg_ of SOL fibers was negative and significantly less than that of both DDF (*P *=**0.01) and SDF (*P *=**0.02) fibers. At 0.02 strain and greater, maximum net *W*_avg_ was either zero or negative in DDF, SDF, and SOL, and there were no significant differences among means for DDF, SDF, and SOL fibers at any high strain amplitude. Figure 4 shows maximum net oscillatory power as a function of frequency for DDF, SDF, and SOL fibers at low strains. Peaks of maximum oscillatory power (expressed relative to cycling frequency) were generally broader for DDF fibers, extending over a wider range of frequencies than for SDF and SOL fibers. On average, maximum oscillatory power occurred at cycling frequencies of 6–7 Hz for DDF, 4–5 Hz for SDF, and 1 Hz for SOL.

**Figure 5.**
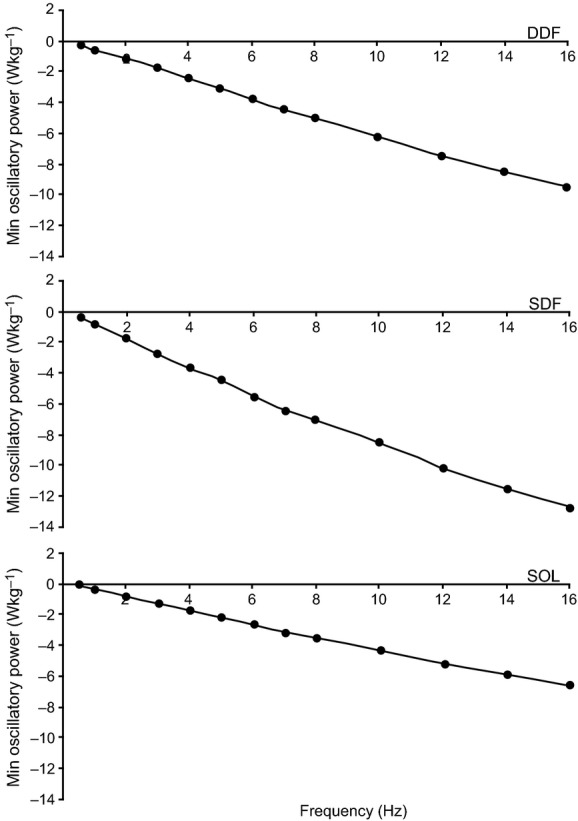
Minimum *net* oscillatory power versus cycling frequency for DDF (top), SDF (middle), and SOL (bottom) fibers at 0.06 strain and 30°C. Oscillatory power was determined by integration of the area within the workloops. Representative data from a single fiber for each muscle is shown.

Means (± SE) of minimum net *W*_avg_ for maximally activated fibers are presented in Table 3. Fibers absorbed relatively larger amounts of power at larger strain amplitudes. Means of minimum net *W*_avg_ among DDF, SDF and SOL fibers were not significantly different at any strain amplitudes (*P *=**0.17–0.88). Figure 5 shows minimum net oscillatory power as a function of frequency for DDF, SDF, and SOL fibers at high strain. Minimum oscillatory power showed a similar pattern with frequency in fibers from each muscle where power absorption increased linearly over the range of cycling frequencies studied.

**Table 3.** Grand means ± SE of minimum net oscillatory power measured by workloop analysis from fibers subject to sinusoidal oscillations and maximal activation (pCa 5; 30°C) for the DDF, SDF, and SOL

Strain	DDF				SDF				SOL			
	Power (W kg^−1^)	*n* (cycles)	Frequency ^ƒ^ (Hz)	*n*	Power (W kg^−1^)	*n* (cycles)	Frequency ^ƒ^ (Hz)	*n*	Power (W kg^−1^)	*n* (cycles)	Frequency ^ƒ^ (Hz)	*n*
0.01	−0.37 ± 0.22	18	16	2	−0.46 ± 0.10	27	16	3	−0.48 ± 0.15	27	16	3
0.02	−0.77 ± 0.34	27	16	3	−1.61 ± 0.55	36	16	4	−2.32 ± 0.52	27	14	3
0.03	−5.00 ± 1.42	18	16	2	−3.04 ± 0.33	27	16	3	−4.06 ± 1.34	18	16	2
0.04	−5.86 ± 1.47	27	16	3	−4.17 ± 0.42	18	16	2	−3.76 ± 0.61	18	16	2
0.05	−10.7 ± 3.37	18	16	2	−7.76 ± 1.10	27	16	3	−6.68 ± 1.83	18	16	2
0.06	−12.0 ± 2.91	36	16	4	−11.4 ± 0.00	9	14	1	−6.54 ± 0.21	27	16	3

ƒ frequency at which most power is absorbed.

*n* (cycles) is the total number of cycles per fiber over which oscillatory power was measured and averaged (W_avg_).

*n* is the number of fiber experiments.

### FFT analysis

**Figure 6.**
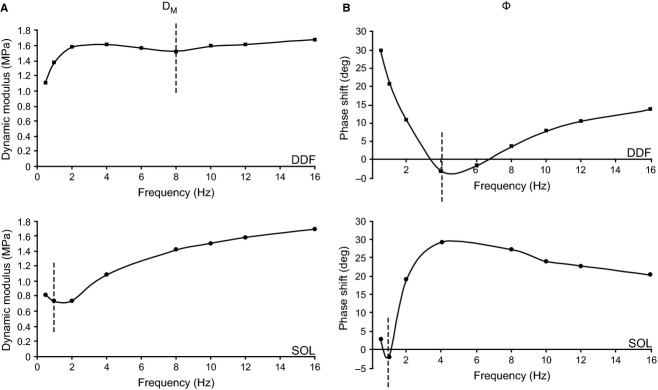
Dynamic modulus (D_M_) and phase shift (*ϕ*) of fast DDF fibers (top panels) and slow SOL fibers (bottom panels) at 0.01 strain and 30°C. (A) *D*_M_ versus frequency, with characteristic minima marked by vertical dashed lines. (B) *ϕ* versus frequency with characteristic minima marked by vertical dashed lines. Representative data from a single fiber for each muscle is shown.

A representative relationship between D_M_ and cycling frequency for maximally activated DDF and SOL fibers at 0.01 strain is shown in Figure 6A. The typical pattern for DDF and SDF fibers was an initial increase in D_M_ at low cycling frequencies, followed by a decrease at intermediate cycling frequencies, and then D_M_ increased again with the higher frequencies. For SOL fibers there was an initial decrease with low cycling frequencies, after which D_M_ increased steadily reaching values greater than 2x the minimum. There was a characteristic minima in D_M_ for SOL commonly at 1 Hz. For DDF and SDF the inflection minimum was right shifted, occurring between 6–12 Hz and 6–8 Hz, respectively. Figure 6B shows the relationship for *ϕ* as a function of cycling frequency at 0.01 strain. The values of *ϕ* initially fall to a minimum (negative angle) and then rise again with increasing frequency in fibers from each muscle. For both DDF and SDF fibers, the characteristic minimum *ϕ* was right shifted to higher frequencies compared with SOL. Frequency of the minimum *ϕ* was 4–7 Hz for DDF, 4 Hz for SDF, and 0.5–1 Hz for SOL.

**Figure 7.**
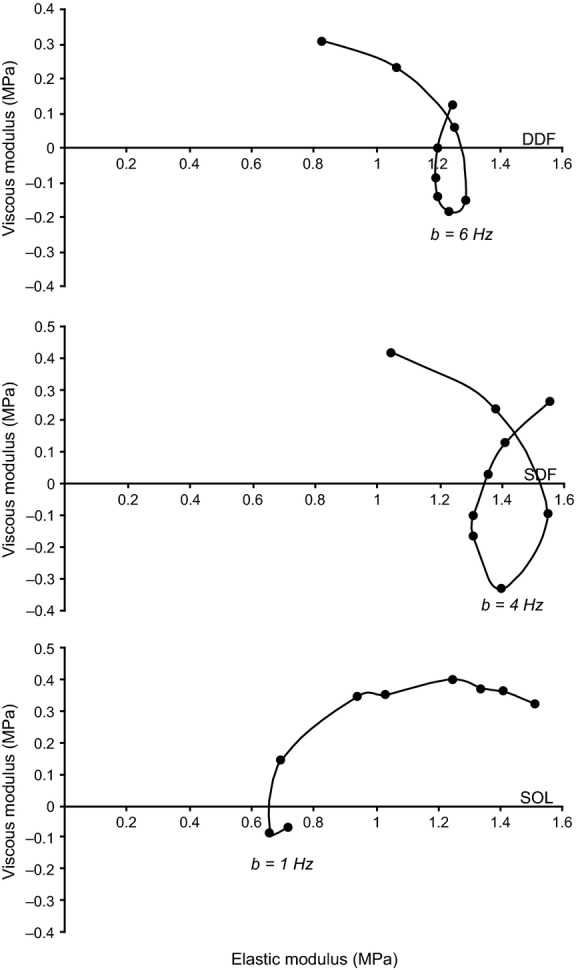
Nyquist plots of maximally activated (pCa 5) DDF (top), SDF (middle), and SOL (bottom) fibers at 0.01 strain and 30°C; data from 2–3 fibers from each muscle are averaged (vertical and horizontal SE bars for each data point are not shown for clarity of presentation). The ordinate is the viscous modulus (*V*: imaginary component) and the abscissa is the elastic modulus (*E*: real component). All data were corrected against rigor (see Materials and Methods). Each data point represents a different cycling frequency, from left‐to‐right (clockwise): 0.5, 1, 2, 4, 6, 8, 10, 12, 16 Hz. Characteristic frequency (*b*) is the frequency at which maximum oscillatory work production occurs (i.e., when *V* is most negative) (see ref. Knuth et al. 2006).

Examples of Nyquist plots for DDF, SDF, and SOL fibers at 0.01 strain are shown in Figure 7. Similar to workloop analysis, Nyquist plots for DDF and SDF fibers showed only small differences between muscles, while both differed substantially from SOL fibers. Nyquist plots for all three muscles showed a portion where *V* was negative (i.e., negative *ϕ*_c_) indicating that oscillatory work was being performed by active cross‐bridges. This occurred over a range of intermediate frequencies, reaching a minimum (i.e., most work done) at a characteristic frequency (b) of 6 Hz for DDF, 4 Hz for SDF, and 1 Hz for SOL, demonstrating differences in oscillatory frequency for power performance in each muscle. Furthermore, Nyquist plots for SOL at 0.01 strain were left shifted on the elastic modulus axis compared with DDF and SDF, reflecting smaller elasticity of SOL fibers associated with lower force development.

At frequencies >1 Hz, SOL fibers showed positive *V* and increasing *E*, such that power was being increasingly absorbed as cycling frequency increased. In contrast, Nyquist plots for DDF and SDF fibers showed distinct loops in the negative region of the viscous modulus axis, where fibers from each muscle are generating positive *net* work and power. The upper range of cycling frequencies where oscillatory work could be performed was greater in DDF than in SDF fibers.

## Discussion

One objective of this study was using power output of muscle fibers to predict whole muscle function of the equine DDF and SDF. Unique to this goal was the use of workloop analysis to quantify the capacity of single, skinned fibers to generate mechanical power at temperatures near physiological. Conventional in vitro workloop analysis (Josephson 1985) on whole, living muscle preparations differs markedly from the techniques used here on skinned fibers, particularly with regard to activation. While experiments on living muscle requires optimization of activation parameters in addition to both strain amplitude and cycling frequency for maximum work (Barclay 1994; James et al. 1995; Askew et al. 1997, 2001; Ashley‐Ross and Barker 2002; Syme et al. 2005), we employed continuous activation in high Ca^2+^ in permeabilized fibers to assess how properties inherent to the cross‐bridges impact their ability to either absorb or generate power, and the cycling frequencies at which they are most effective. For these reasons values of work and power derived from living muscle versus permeabilized fibers, and likely the frequencies at which they are maximized, may not be directly comparable and are not discussed here.

At strain amplitudes of 0.01 *L*_0_, fibers from the DDF and SDF, but not the SOL, show a capacity to generate oscillatory work and power in the conditions used for these experiments. These observations are consistent with previous studies done at low strain amplitudes on mammalian skeletal muscle fibers (Kawai and Brandt 1980) and whole fibrillar insect flight muscle from rhinoceros beetles (Machin and Pringle 1959, 1960). Overall, DDF and SDF fibers generated similar amounts of power at low strain amplitude and cycling frequency. This finding was unexpected in light of our previous results showing a higher *P*_o_, *V*_US_, and percentage of fast MHC‐2A fibers in the DDF (83%) compared with the SDF (48%) (Butcher et al. 2010). Although MHC isoforms were not identified in this study, work and power of DDF and SDF fibers can be clearly distinguished from the SOL that has a 100% slow MHC‐1 composition (Butcher et al. 2010), where the frequency dependent relationships of maximum oscillatory power for DDF and SDF fibers show peaks in power at distinctly faster frequencies compared with SOL fibers (Figs. 4,7). Thus, the presence of the fast MHC‐2A isoform appears to convey a capacity to generate more power than fibers expressing MHC‐1 only, and greater percentages of MHC‐2A fibers can be associated with higher frequencies at which maximal power is generated.

Fibers from the DDF, SDF, and SOL all had a relatively large capacity for oscillatory work and power absorption. The slope of the force‐length ellipses generated when sinusoidal strain was imposed on the activated fibers (Fig. 3) became progressively steeper with increasing frequency at a given strain, suggesting an increase in stiffness of the fibers with higher frequencies, as would be expected for viscoelastic muscle fibers that absorb energy. Oscillatory power absorption is of interest with respect to function of the specialized equine digital flexors during locomotion. Based on muscle model predictions (Wilson et al. 2001), it has been argued that the SDF (and DDF, humeral long compartment; see ref. Hermanson and Cobb 1992) fascicles are well developed for damping high frequency (30–100 Hz), small displacement vibrations of the forelimb following hoof impact. The highest fiber oscillatory frequency used in the present study was 16 Hz and therefore, values of energy absorption between 30–100 Hz cannot be experimentally verified with our data. However, the pattern of increasing energy absorption with increasing cycle frequency (Fig. 5) suggest a potentially large capacity for damping by the DDF and SDF (relatively faster fibers), and SOL (slow fibers), at the frequencies observed in running horses. The SDF is noted for its potential role as an energy absorber (Butcher et al. 2009) and this reduces metabolic energy consumption. At the relatively large strains typical during locomotion [recorded fascicle strains in vivo in the SDF show peak strain of 3.5% during fast trotting (ref. Butcher et al. 2009)], SDF fibers had an appreciable capacity for power absorption, even at oscillation frequencies well below those expected after a footfall. Thus, these findings appear to support the previous conclusions (Wilson et al. 2001), that the SDF functions to damp vibrations and support the body weight (Butcher et al. 2010). However, the assertion that robust muscle fibers are indicative of a muscle architecture for damping high frequency vibrations within the limb is not supported by the findings of the present study. Thin SOL fibers (i.e., small fiber CSA) appear to have an equally large capacity to absorb power as DDF and SDF fibers at strain amplitudes up to 0.05 *L*_0_ (Table 3; Fig. 5).

A clear distinction between muscles can be made when comparing the characteristic frequency (*b*) for maximum oscillatory work from the Nyquist plots. The characteristic frequency of horse slow SOL fibers for maximum oscillatory work (Fig. 7) was the lowest of the three muscles, and in close agreement with *b *=**0.64 Hz reported for slow rabbit soleus fibers at 20°C (Wang and Kawai 1996). DDF fibers had the fastest *b* and a higher range of cycling frequencies over which oscillatory power was generated (i.e., negative *V*) compared with either SDF or SOL fibers, as is typical of a muscle composed of a high percentage of fast fibers. At strain amplitude 0.01 *L*_0_, the *V* for DDF fibers was negative between 4–10 Hz, SDF fibers performed oscillatory work between 2–8 Hz, and SOL only at 0.5–1 Hz. Comparatively, MHC‐2X fibers of rabbit psoas have characteristic frequencies *b *=**14–19 Hz at strains of 0.0025–0.005 at 20°C (Zhao and Kawai 1994; Murphy et al. 1996), while MHC‐2A fibers of rabbit soleus have a slower *b *=**6.3 Hz under similar experimental conditions (Wang and Kawai 1996). It is interesting that the characteristic frequency *b *=**6 Hz for horse DDF fibers (~80% MHC‐2A) at 30°C is nearly equal to that of MHC‐2A fibers from the rabbit at 20°C (Wang and Kawai 2001), which raises the issue of scaling of muscle fiber contractile kinetics across body size. It is well established that *V*_0_ (or *V*_US_) for each MHC isoform fiber type scales negatively with body size across a broad range of mammalian species (Seow and Ford 1991; Toniolo et al. 2007). Mass‐specific power output of muscle fibers has also been shown to scale inversely with body mass (Seow and Ford 1991; Pellegrino et al. 2003), although similar in vivo power estimates for quail (Askew et al. 2001), lizard (Curtin et al. 2005), and cheetah muscle fibers (West et al. 2013) indicate this relationship requires further investigation using similar methods at or near physiological temperature. The slower *b* of horse muscle compared with rabbit muscle of the same fiber type, and the slower characteristic frequency of SOL versus SDF versus DDF fibers, are in accordance with expectations of these scaling relationships, predicting a lower range of cycle frequencies over which muscle will be able to produce power in larger animals or slower muscles.

As discussed for workloop analysis, the small differences between DDF and SDF Nyquist plots is likely the result of similarity in the MHC isoforms expressed in fibers randomly selected for study. This would also explain their dissimilarity with SOL fibers of MHC‐1 isoform composition. Comparison with Nyquist plots from rabbit fibers of known MHC and myosin light chain (MLC) isoform composition further support this interpretation (Kawai and Schachat 1984). Therefore, the qualitative shape of the Nyquist plot can be a sensitive tool for determining whether a fiber is of “fast” or “slow” type (Kawai and Brandt 1980) and complements other physiological measurements distinguishing muscle fiber type such as filament sliding velocity (*V*_f_) and velocity of unloaded shortening (*V*_us_) (Butcher et al. 2010), and force‐velocity properties. The relatively fast fiber type distributions of DDF and SDF compared with SOL will also have important energetic consequences that can be assessed by Nyquist analysis. The larger loops (oscillatory work production) of DDF and SDF fibers compared with SOL (Fig. 3) are directly related to faster rates of ATP hydrolysis (Rüegg and Tregear 1966), and the faster *b* reflects faster rate constants of biochemical reactions involved in the transduction steps of the cross‐bridge power stroke (i.e., Process B of ref. Kawai and Brandt 1980). Slower rate constants for MHC‐1 fibers is suggestive of slower rates of cross‐bridge cycling and possibly fewer cross‐bridges (Wang and Kawai 1996, 1997), a reasonable suggestion for a fiber type with a lower capacity for power output. However, it is important to note that the values of *b* identified in this study are not necessarily the same as the cross‐bridge model parameter estimates previously obtained (Murphy et al. 1996; Wang and Kawai 1996, 1997, 2001) because these analyses necessitate variations in the concentrations of ATP, Pi, and ADP. In particular, the baseline condition for analyses of cross‐bridge dynamics has relatively high [Pi] to yield Nyquist plots with large (i.e., open) work‐producing regions (Kawai and Brandt 1976, 1980; Kawai and Schachat 1984; Kawai 1986).

Lastly, analysis of D_M_ and *ϕ* used to construct Nyquist plots can also be used to assess fiber type contractile properties. There was a higher frequency at which the characteristic minima of both D_M_ and *ϕ* occurred for DDF and SDF fibers compared with SOL fibers (Fig. 6), indicating faster apparent rate constants, and thus faster cross‐bridge kinetics (Wang and Kawai 1996, 1997). Similar findings were made comparing MHC‐1 fibers from rabbit soleus, semitendinosus, and diaphragm muscles with fast fiber types from psoas and EDL muscles (Kawai and Schachat 1984), where the characteristic minimums of slow fibers were shifted to slower frequencies by nearly 30‐fold, comparable to the findings for SOL fibers in our study. A final important point is that increases in temperature will shift the frequency of the characteristic minima of D_M_ and *ϕ* toward higher values. It has been clearly shown that over a range of temperatures up to physiological, a distinct right shift in D_M_ and *ϕ* versus frequency relationships occurs for both fast (Zhao and Kawai 1994) and slow (Wang and Kawai 2001) fiber types of the rabbit with increasing temperature. The D_M_ and *ϕ* frequency relationships at 30°C are likely representative of physiological differences between DDF, SDF, and SOL fibers, and suggest that DDF and SDF fibers share similar cross‐bridge dynamics that are distinct from and much faster than purely slow SOL fibers.

## Summary and Conclusions

Oscillatory work and power measured in maximally activated, skinned fibers at 30°C were similar for the DDF and SDF. However, fast fibers from the DDF generated power at higher frequencies, and both DDF and SDF produced more power and at much higher frequencies than fibers from the slow SOL. Nyquist plots confirmed slightly higher characteristic frequencies for oscillatory work for the DDF, and further demonstrated a capacity for DDF fibers to perform oscillatory work over a higher range of frequencies than SDF fibers. These results are consistent with our hypothesis that higher frequencies for oscillatory work and power generation are associated with faster cross‐bridge kinetics that are characteristic of fast MHC isoforms expressed in horse muscles. However, our result that the slow SOL appears capable of substantial power absorption, even at relatively high frequencies, does not support the contention that muscles with faster (and larger fiber CSA) fibers are essential to perform this role. Based on these collective findings, we conclude that the fast DDF muscle will have a greater capacity to generate power than the SDF, and fibers from both muscles are capable of considerable energy absorption, which may be important to the energetics of horse locomotion.

To place the findings of this study in a broader functional and evolutionary context, the ability to shorten and generate power would be further enhanced in the intact DDF muscle by its long‐fibered, unipennate muscle architecture (Butcher et al. 2009), and this matches the mechanical work performance of the DDF (short compartment) to flex the metacarpophalangeal joint during running (Butcher et al. 2009). In contrast, the multipennate SDF muscle has very short fibers and undergoes high force, lengthening contractions in vivo during locomotion that absorb work and power, and reduce metabolic energy consumption in running horses (Butcher et al. 2007, 2009). The evolution of this extremely specialized muscle architecture would constrain the ability of its fibers to generate power during running, even though individual muscle fibers appear capable of producing power at relatively high frequencies. Therefore, we further suggest that function of DDF and SDF muscles may be much more dependent on their fiber architecture in the living animal than on the specific physiological properties of their muscle fibers. High power muscles in horse forelimbs may have been selected against in favor of lower power muscle fibers that conserve energy for long distance running.

## Acknowledgments

The authors thank the Cornell University, College of Veterinary Medicine, Department of Necropsy for access to horses for muscle tissue biopsies. We sincerely thank Dr. A. Kataoka Takeda, Dr. N. Brunet, A. Clark, and J. Bhuvasorakul for assistance with fiber data sorting and analyses. A special thanks to J. Rose (YSU) for construction of the final figures. Portions of this work were submitted as a doctoral dissertation at the University of Calgary (U of C) by MTB. Preparation of this manuscript was permitted by teaching reassignment time awarded to MTB at Youngstown State University (YSU). The YSU, FSU, and U of C Departments of Biological Science(s) are also gratefully acknowledged.

## Conflict of Interest

The authors declare no conflicts of interests.
